# Anti-Human Rhinoviral Activity of Polybromocatechol Compounds Isolated from the Rhodophyta, *Neorhodomela aculeata*

**DOI:** 10.3390/md10102222

**Published:** 2012-10-10

**Authors:** Soon-Hye Park, Jae-Hyoung Song, Taejung Kim, Woon-Seob Shin, Gab Man Park, Seokjoon Lee, Young-Joo Kim, Pilju Choi, Heejin Kim, Hui-Seong Kim, Dur-Han Kwon, Hwa Jung Choi, Jungyeob Ham

**Affiliations:** 1 Marine Chemomics Lab., Natural Medicine Center, Korea Institute of Science and Technology, Gangneung 210-340, Korea; Email: psh@kist.re.kr (S.-H.P.); kgsing@kist.re.kr (T.K.); zoo@kist.re.kr (Y.-J.K.); 022450@kist.re.kr (P.C.); heejinmail@kist.re.kr (H.K.); 2 Zoonosis Research Center, Wonkwang University College of Medicine & Oriental Medicine, 344-2, Iksan, Chonbuk 570-749, Korea; Email: thdwohud@naver.com; 3 Department of Microbiology and Basic Science, Kwandong University College of Medicine, Gangneung 210-701, Korea; Email: shinws@kd.ac.kr (W.-S.S.); gmpark@kd.ac.kr (G.M.P.); sjlee@kd.ac.kr (S.L.); 4 Immune Modulator Research Center, Korea Research Institute of Bioscience and Biotechnology, Daejeon 305-333, Korea; Email: fungdori@kribb.re.kr (H.-S.K.); dhkwon@kribb.re.kr (D.-H.K.)

**Keywords:** *Neorhodomela aculeate*, red alga, polybromocatechol compounds, antiviral activity, human rhinovirus

## Abstract

An extract of the red alga, *Neorhodomela aculeata*, exhibited antiviral activity against human rhinoviruses. Bioassay-guided purification was performed to yield six compounds, which were subsequently identified as lanosol (**1**) and five polybromocatechols (**2**–**6**) by spectroscopic methods, including 1D and 2D NMR and mass spectrometric analyses. Structurally, all of these compounds, except compound **5**, contain one or two 2,3-dibromo-4,5-dihydroxyphenyl moieties. In a biological activity assay, compound **1** was found to possess antiviral activity with a 50% inhibitory concentration (IC_50_) of 2.50 μg/mL against HRV2. Compound **3** showed anti-HRV2 activity, with an IC_50_ of 7.11 μg/mL, and anti-HRV3 activity, with an IC_50_ of 4.69 μg/mL, without demonstrable cytotoxicity at a concentration of 20 μg/mL. Collectively, the results suggest that compounds **1** and **3** are candidates for novel therapeutics against two different groups of human rhinovirus.

## 1. Introduction

More than 500 species of marine algae are distributed along the coast of Korea. Some of these algae are sources of foods or traditional medicines [1], while others may serve as important resources for bioactive natural products [2,3,4]. Red algae of the Rhodomelaceae (Ceramiales) family are rich sources of several monoaryl, diaryl, and triaryl bromocatechol structural types with various biological activities, including anticancer, antioxidative, antimicrobial, and anti-thrombotic effects [5,6,7,8,9]. However, there have been no reports describing the characterization of compounds derived from *Neorhodomela aculeata *(L.P. Perestenko) Masuda. (Rhodomelaceae) collected off the Korean coast. A few reports have described the antioxidative and antibacterial activities of methanolic extracts of *N. aculeata*, but there have not been reports of any antiviral activity associated with these extracts [10].

Human rhinoviruses (HRVs), members of the Picornaviridae family, are divided into three distinct species: type A, type B, and type C. These viruses are the predominant causal agents of viral respiratory tract infections, particularly common colds [11], as well as acute otitis media and sinusitis [12,13]. Specific and small-molecule antiviral agents for the treatment of picornavirus infections are not currently available. The production of vaccines to prevent rhinovirus infections is also known to be very challenging due to the more than 100 immunologically non-cross-reactive rhinovirus serotypes [14]. Therefore, most efforts have been focused on the development of effective antiviral agents for treating rhinovirus infections [15]. Several antiviral compounds have been shown to inhibit members of the picornavirus family by binding to the viral capsid proteins [16]. One of these, pleconaril, is a new-generation antiviral agent that has shown activity against rhinoviruses, excluding the 25 serotypes of HRV-B [17].

Herein, we report the structurals elucidation of polybromocatechol compounds isolated from *N. aculeata* and their anti-human rhinovirus activity.

## 2. Results and Discussion

### 2.1. Antiviral Activity of *N. aculeata* Extracts and Fractions

The methanol (MeOH) extract of *N. aculeata* was suspended in distilled water and partitioned successively with hexane, ethyl acetate (EtOAc), and *n*-butanol. Each organic fraction was subjected to cytotoxicity and antiviral activity assays (Table 1). Among these, the EtOAc-soluble fraction (ESF) exhibited inhibitory effect against HRV2 and HRV3, with IC_50_ values of 15.50 ± 4.17 μg/mL and 16.50 ± 4.17 μg/mL, respectively. The water soluble fraction (WSF) showed antiviral activity against HRV2, but not against HRV3. The hexane-soluble (HSF) and butanol-soluble (BSF) fractions showed no antiviral activity against either HRV2 or HRV3 (Table 1). 

**Table 1:** Antiviral activity of *Neorhodomela aculeata* extracts against HRV2 and HRV3 in HeLa cells.

**Test material**		**HRV2 (Type B)**		**HRV3 (Type A)**	
	CC_50_^a^	IC_50_^b^	TI ^c^	IC_50_^b^	TI ^c^
Methanolic extract	>20	17.58 ± 0.59	1.14	18.27 ± 2.22	1.09
HSF	>20	ND ^d^	-	ND ^d^	-
ESF	>20	15.50 ± 4.17	1.29	16.50 ± 4.17	1.21
BSF	>20	ND ^d^	-	ND ^d^	-
WSF	>20	16.50 ± 2.76	1.21	ND ^d^	-

Results are presented as mean IC_50_ values obtained from three independent experiments carried out in triplicate ± S.D. ^a^ Concentration required to reduce cell growth by 50% (µg/mL); ^b^ Concentration required to inhibit virus-induced cytopathic effect (CPE) by 50% (µg/mL); ^c^ Therapeutic index = CC_50_/IC_50_; ^d^ IC_50_ value within concentration of the compound to test not determined due to maximum inhibition rate of under 50%.

**Figure 1:**
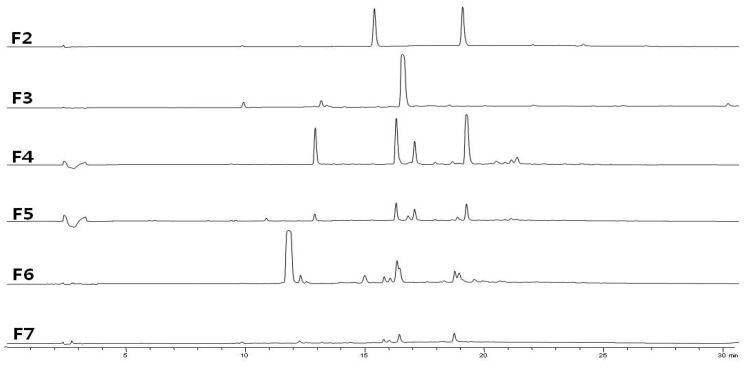
RP-HPLC profile of sub-fractions of EtOAc-soluble fraction (ESF) of *N. aculeata*. Performed on an Agilent 1300 HPLC system fitted with a Phenomenex Luna C18 (2) column (150 × 4.6 mm, 5 μm). The elution solvent system was binary gradient of solvent A (0.02% trifluoroacetic acid (TFA) in water); solvent B (0.02% TFA in acetonitrile). The gradient flow program was, as follows: 0 min, 10% B; 30 min. The flow rate was 0.7 mL/min and detection wavelength was set at 280 nm and column temperature was 25 °C. The chromatogram of F1 was not shown.

The most active fraction, ESF, was fractionated by silica gel column chromatography. Seven fractions (F1–F7) were analyzed by reversed-phase HPLC, monitoring at 280 nm (Figure 1), and tested on HeLa cells for antiviral activity (Table 2).

Fraction 4 (F4) was investigated for its antiviral activity and was demonstrated to have an IC_50_ value of 18.55 ± 0.51 μg/mL against HRV2 and 18.52 ± 0.49 μg/mL against HRV3 (Table 2). Fractions F1, F2, and F7 showed antiviral activity against only HRV2, with IC_50_ values of 11.38 ± 3.01 μg/mL, 8.36 ± 2.42 μg/mL, and 10.69 ± 1.34 μg/mL, respectively. F3 only showed antiviral activity against HRV3, with a IC_50_ value of 7.69 ± 0.45 μg/mL (Table 2).

**Table 2:** Antiviral activity of sub-fractions of ESF against HRV2 and HRV3 in HeLa cells.

**Test material**	**HRV2**			**HRV3**		
	CC_50_^a^	IC_50_^b^	TI ^c^	CC_50_^a^	IC_50_^b^	TI ^c^
F1	>20	11.38 ± 3.01	1.76	>20	ND ^d^	-
F2	>20	8.36 ± 2.42	2.39	>20	ND ^d^	-
F3	22.93	ND ^d^	-	25.80	7.69 ± 0.45	2.60
F4	>20	18.55 ± 0.51	1.08	>20	18.52 ± 0.49	1.08
F5	24.29	ND ^d^	-	18.98	ND ^d^	-
F6	>20	ND ^d^	-	>20	ND ^d^	-
F7	>20	10.69 ± 1.34	1.87	>20	ND ^d^	-
Ribavirin	>20	17.14 ± 1.48	1.17	>20	14.25 ± 2.20	1.40

Results are presented as mean IC_50_ values obtained from three independent experiments carried out in triplicate ± S.D. ^a^ Concentration required to reduce cell growth by 50% (µg/mL); ^b^ Concentration required to inhibit virus-induced cytopathic effect (CPE) by 50% (µg/mL); ^c^ Therapeutic index = CC_50_ / IC_50_; ^d^ IC_50_ value within concentration of the compound to test not determined due to maximum inhibition rate of under 50%.

**Figure 2:**
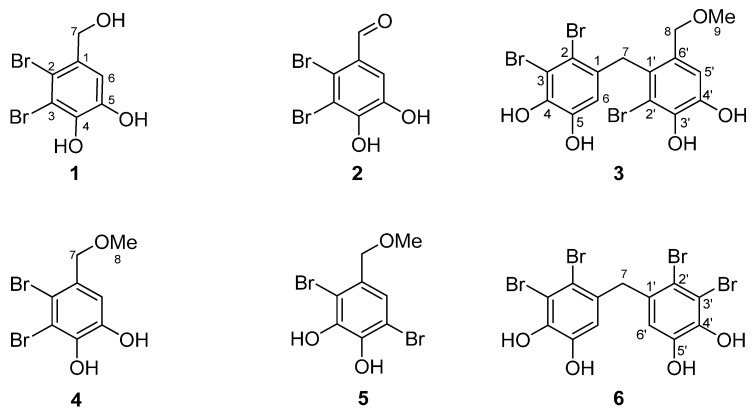
Structures of compounds **1**–**6**.

Fraction F4 was purified by preparative HPLC and semi-preparative HPLC at 280 nm, affording six pure compounds (**1**–**6**), as shown in Figure 2. Their structures were elucidated by spectroscopic methods, including 1D and 2D NMR techniques and MS. 

Compound **1** was a yellowish-brown needle-shaped crystal, with a molecular formula of C_7_H_5_^79^Br^81^BrO_3_, as determined from ^13^C data (Table 3) and ESIMS *m/z* 296.9 [M − H]^−^. The ^1^H NMR spectrum of compound **1** in acetone-*d*_6_ showed the presence of a single aromatic proton at δ_H_ 7.20 (1H, s, H-6) and a methylene group at δ_H_ 4.62 (2H, s, H-7) on a catechol. The ^13^C signals at δ_C_ 144.8, 143.1, 134.1, 113.7, 112.6, 112.4, and 64.2 corresponded to carbons C-5, C-4, C-1, C-6, C-2, C-3, and C-7, respectively. Accordingly, the structure of **1** was determined to be 2,3-dibromo-4,5-dihydroxybenzyl alcohol (lanosol) (Table 3) [18].

Compound **2** was a colorless solid with a molecular formula of C_7_H_5_^79^Br^81^BrO_3,_ as determined by ESIMS *m/z* 296.9 [M + H]^+^. The ^1^H NMR spectrum of compound **2** in acetone-*d*_6_ exhibited an aldehyde proton signal at δ_H_ 10.22 (1H, s, H-7) and an aromatic proton signal at δ_H_ 7.41 (1H, s, H-6). Six aromatic carbon signals were observed at δ_C_ 157.0 (C-4), 150.3 (C-5), 144.4 (C-4), 127.4 (C-3), 120.7 (C-2), and 113.1 (C-6), and an aldehyde carbon signal was seen at δ_C_ 190.4 (C-7). Therefore, the structure of **2 **was determined to be 2,3-dibromo-4,5-dihydroxybenzaldehyde (Table 3) [19]. 

Compound **3** was also obtained as a colorless solid having a molecular formula of C_15_H_13_^79^Br_2_^81^BrO_5_Na, as determined by ESIMS *m/z* 534.8 [M + Na]^+^. In addition to a singlet exchangeable signal that integrated for four protons at δ_H_ 8.73 (s, 1H, OH-4), 8.71 (s, 1H, OH-3′), 8.17 (s, 1H, OH-4′), and 8.08 (s, 1H, OH-5); the ^1^H NMR spectrum of the compound in acetone-*d*_6_ demonstrated two singlets attributable to aromatic protons at δ_H_ 7.00 (s, 1H, H-5′) and 6.08 (s, 1H, H-6), two methylene groups at δ_H_ 4.21 (s, 2H, H-8) and 4.12 (s, 2H, H-7) and a methoxy group at δ_H_ 3.24 (s, 3H, H-9). The ^13^C NMR spectra of compound **3 **showed 15 carbons, assignable to two benzene rings at δ_C_ 115.4 (C-5′), and 114.0 (C-6); two methylenes at δ_C_ 72.3 (C-8) and 38.4 (C-7); and a methoxy at δ_C_ 57.3 (C-9) with four oxygenated carbons at δ_C_ 144.5 (C-4′), 144.0 (C-5), and 142.6 (C-4, C-3′). The above spectral data indicated that compound **3 **possessed a tribrominated diphenylmethane structure with four hydroxyl groups and one methoxy group. Thus, the structure of **3 **was determined to be 2,2′,3-tribromo-3′,4,4′,5-tetrahydroxy-6′-methoxymethyldiphenylmethane (Table 3) [20].

Compound **4** was also isolated as a colorless solid, but with a molecular formula of C_8_H_7_^79^Br^81^BrO_3_, as determined by ESIMS *m/z* 310.9 [M − H]. The ^1^H NMR spectrum of compound **4** in acetone-*d*_6_ showed one singlet aromatic proton at δ_H_ 7.07 (s, 1H, H-6); a methylene group at δ_H_ 4.41 (s, 2H, H-7) of a catechol; and an attached methoxy at δ_H_ 3.39 (s, 3H, H-8). It was also determined to have five aromatic carbon signals at δ_C_ 144.9 (C-5), 143.7 (C-4), 131.6 (C-1), 114.8 (C-2, 6), and 114.4 (C-3); one methylene carbon signal at δ_C_ 75.0 (C-7); and one methoxy carbon signal at δ_C_ 58.4 (C-8). Therefore, the structure of **4 **was determined to be 2,3-dibromo-4,5-dihydroxybenzyl methyl ether (Table 3) [18].

Compound **5 **was another colorless solid, with an ESIMS *m/z* 310.9 [M − H]-determined molecular formula of C_8_H_7_^79^Br^81^BrO_3_. The ^1^H NMR spectrum of the compound in acetone-*d*_6_ revealed the presence of one aromatic hydrogen at δ_H_ 6.71 (s, 1H, H-6); a methylene group at δ_H_ 4.43 (s, 2H, H-7) of a catechol; and an attached methoxy at δ_H_ 3.34 (s, 3H, H-8). The compound also showed six aromatic carbon signals at δ_C_ 152.2 (C-4), 144.9 (C-3), 129.1 (C-1), 112.0 (C-2), 111.9 (C-6), 109.3 (C-5); one methylene carbon signal at δ_C_ 75.2 (C-7); and one methoxy carbon signal at δ_C_ 57.9 (C-8). Thus, the structure of **5 **was established as 2,5-dibromo-3,4-dihydroxy-benzyl methyl ether (Table 3) [18].

Compound **6** was purified as a white amorphous solid with a molecular formula of C_13_H_7_^79^Br_2_^81^Br_2_O_4_, determined using ESIMS *m/z* 546.7 [M − H]. Its ^1^H NMR spectrum revealed one aromatic hydrogen at δ_H_ 6.58 (s, 2H, H-6, 6′) and a methylene group at δ_H_ 4.04 (s, 2H, H-7). The ^13^C NMR spectra showed seven carbons assignable to one benzene ring at δ_C_ 145.4 (C-5, 5′), 143.8 (C-4, 4′), 132.0 (C-1, 1′), 116.5 (C-6, 6′), 116.4 (C-2, 2′), and 113.6 (C-3, 3′), and to one methylene group at δ_C_ 44.5 (C-7). Consequently, the assigned structure was 2,2′,3,3′-tetrabromo-4,4′,5,5′-tetrahydroxydiphenyl methane (Table 3) [20].

**Table 3:** The ^1^H and ^13^C NMR spectroscopy data of compounds **1**–**6**^a^.

No.		1			2			3			4			5			6	
		δ_H_	δ_C_		δ_H_	δ_C_		δ_H_	δ_C_		δ_H_	δ_C_		δ_H_	δ_C_		δ_H_	δ_C_
**1**			134.1			144.4			131.5			131.6			129.1			132.0
**2**			112.6			127.4			115.4			114.8			112.0			116.4
**3**			112.4			120.7			112.8			114.4			144.9			113.6
**4**			143.1			157.0		8.73 (s, 1H, OH)	142.6			143.7			152.2			143.8
**5**			144.8			150.3		8.08 (s, 1H, OH)	144.0			144.9			109.3			145.4
**6**		7.20 (s, 1H)	113.7		7.41 (s, 1H)	113.1		6.08 (s, 1H)	114.0		7.07 (s, 1H)	114.8		6.71 (s, 1H)	111.9		6.58 (s, 1H)	116.5
**7**		4.62 (s, 2H)	64.2		10.22 (s, 1H)	190.4		4.12 (s, 2H)	38.4		4.41 (s, 2H)	75.0		4.43 (s, 2H)	75.2		4.04 (s, 2H)	44.5
**8**								4.21 (s, 2H)	72.3		3.39 (s, 3H)	58.4		3.34 (s, 3H)	57.9			
**9**								3.24 (s, 3H)	57.9									
**1′**									128.6									132.0
**2′**									114.0									116.4
**3′**								8.71 (s, 1H, OH)	142.6									113.6
**4′**								8.17 (s, 1H, OH)	144.5									143.8
**5′**								7.00 (s, 1H)	115.4									145.4
**6′**									129.8								6.58 (s, 1H)	116.5

^a^ The NMR data were measured in acetone-*d*_6_ (δ_H_ 2.05 ppm; δ_C_ CO 205.8, CH_3_ 30.6 ppm) at 500 MHz for ^1^H and at 125 MHz for ^13^C.

**Table 4:** Antiviral activity of compound **1** and compound **3** isolated from *N. aculeata *against HRV 2 and HRV3.

**Test material**		**HRV2**		**HRV3**	
	CC_50_^a^	IC_50_^b^	TI ^c^	IC_50_^b^	TI ^c^
Compound **1**	>20	2.50 ± 0.66	8.00	ND ^d^	-
Compound **3**	>20	7.11 ± 0.64	2.81	4.69 ± 0.44	4.26
Ribavirin	>20	2.15 ± 0.51	9.30	5.09 ± 0.60	3.93

Results are presented as mean IC_50_ values obtained from three independent experiments carried out in triplicate ± S.D. ^a^ Concentration required to reduce cell growth by 50% (µg/mL); ^b^ Concentration required to inhibit virus-induced cytopathic effect (CPE) by 50% (µg/mL); ^c^ Therapeutic index = CC_50_/IC_50_; ^d^ IC_50_ value within concentration of the compound to test not determined due to maximum inhibition rate of under 50%.

### 2.2. Antiviral Activity and Cytotoxicity of Compound ***1*** and Compound ***3*** against HRV2 and HRV3

The antiviral activity of the six isolated compounds (**1**–**6**) was tested; compounds **2**, **4**, **5**, and **6** showed no antiviral effect (data not shown). However, the antiviral assays demonstrated that compound **1** showed anti-HRV2 activity with a 50% inhibitory concentration (IC_50_) value of 2.50 μg/mL and a 50% cytotoxic concentration (CC_50_) value of more than 20 μg/mL, although it did not show anti-HRV3 activity (Table 4). Compound **3** also possessed strong antiviral activity with IC_50_ values of 7.11 μg/mL against HRV2 and 4.69 μg/mL against HRV3, and a CC_50_ value of more than 20 μg/mL (Table 4). Ribavirin, tested as a positive control, also showed antiviral activity in HeLa cells infected with HRV2 and HRV3 with IC_50_ values of 2.15 μg/mL and 5.09 μg/mL, respectively, and exhibited a CC_50_ value of more than 20 μg/mL (Table 4).

**Figure 3:**
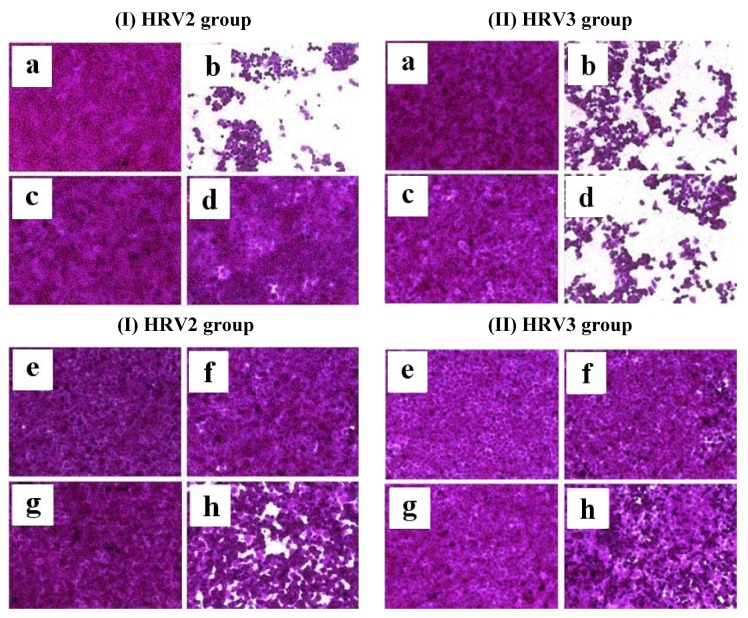
The effect of compound **1** or compound **3** on HRV2 (left group; **I**) and HRV 3 (right group; **II**)-induced CPE. The virus-infected cells were treated with compound **1** or compound **3** of 20 μg/mL. After incubation at 32 °C in 5% of CO_2_ for 2 days, the cell morphology was photographed under a microscope. (**a**) Non-infected cells; (**I-b**) HRV2 or (**II-b**) HRV3-infected cells without compound; (**c**) non-infected cells treated with compound **1**; (**I-d**) HRV2 or (**II-d**) HRV3-infected cells with compound **1**; (**e**) non-infected cells treated with compound **3**; (**I-f**) HRV2 or (**II-f**) HRV3-infected cells with compound **3**; (**g**) non-infected cells treated with ribavirin; (**I-h**) HRV2 or (**II-h**) HRV3-infected cells with ribavirin.

After a 2-day infection of HeLa cells with HRV2 and HRV3, the effect of compound **1** and compound **3** on HRV-induced cytopathic effect (CPE) investigated. As the results, uninfected cells (Figure 3-I-a) or cells treated with compound **1** (Figure 3-I-c), compound **3** (Figure 3-I-e), and ribavirin (Figure 3-I-g) showed typical spread-out shapes and normal morphology. No signs of cytotoxicity were observed for compounds **1**, **3,** or ribavirin at exposure concentrations of 20 μg/mL. Infection with HRV2 and HRV3 in the absence of compounds **1** and **3** resulted in a severe CPE (Figure 3-I-b and 3-II-b). The addition of compound **1** or **3** to cultures of infected HeLa cells inhibited the formation of a visible CPE (Figure 3-I-d, 3-I-f, and 3-II-f). Incubation of HRV2- or HRV3-infected HeLa cells with ribavirin also prevented a CPE (Figure 3-I-h and 3-II-h).

## 3. Experimental Section

### 3.1. General Experimental Procedures; Instruments and Reagents

NMR spectra were recorded in acetone-*d*_6_ on a Varian UNITY Plus 500 MHz spectrometer (Palo Alto, CA, USA). NMR chemical shifts were referenced to the residual solvent peaks (δ_H_ 2.05 and δ_C_ 29.84 and 206.26 for acetone-*d*_6_). Low-resolution ESIMS data were measured with an Agilent Technologies VS/Agilent 1100 system (Santa Clara, CA, USA). Stationary phases for column chromatography (Silica gel 60, 230–400 mesh) were purchased from Merck (Darmstadt, Germany). The RP-HPLC system was composed of a solvent degasser (Agilent, G1322A), binary pump (Agilent, G1312C), an autosampler (Agilent, G1329B), and a photodiode-array detector (Agilent, G1315D). Analytical RP-HPLC was used Phenomenex Luna C18 (2) column (150 × 4.6 mm, 5 μm), the mobile phase consisting of a binary gradient of solvent A (0.02% TFA in water); solvent B (0.02% TFA in acetonitrile). The gradient flow program was, as follows: 0 min, 10% B; 30 min, 100% B. The flow rate was 0.7 mL/min and elution was monitored at 280 nm. The active extract was performed in preparative conditions, using a preparative Phenomenex Luna C18 (2) column (250 × 21.2 mm, 10 μm) with a flow rate fixed at 10 mL/min. Purification of the active fraction was performed in semi-preparative conditions, using a semi-preparative Phenomenex Luna C18 (2) column (250 × 10.0 mm, 5 μm) with a flow rate fixed at 4 mL/min. Sulforhodamine B (SRB) was purchased from Sigma-Aldrich (St Louis, MO, USA).

### 3.2. Alga Material

The red alga *Neorhodomela aculeata* was collected in the port of Namae (37°56′43.05″ N, 128°47′13.70″ E), Korea, in July 2006. The sample was frozen when it collected immediately. The specimen identification was verified by Prof. Gab Man Park (Kwandong University). A voucher specimen (KDU-NA, MNP176) was deposited at the Marine Biomedical Research Center, College of Medicine, Kwandong University, Gangneung, 210–701, Korea. 

### 3.3. Extraction and Isolation

*Neorhodomela aculeata* (3 kg, wet wt.) was extracted twice with 100% MeOH at room temperature. The MeOH extract was evaporated to dryness, and then crude residual (3.3 g) was suspended in H_2_O and partitioned successively with hexane, EtOAc and BuOH to give the ESF (2.2 g). Vacuum column chromatography (VCC) (Merck, 230–400 mesh, i.d. 6.5 × 5.0 cm) with *n*-hexane:EtOAc:MeOH (10:1:0, 5:1:0, 1:1:0, 0:100:0, 0:10:1, 0:5:1 and 0:0:100; each 1 L) in a stepwise gradually as eluents. The 7 fractions were combined by VCC and there were performed by analytical HPLC (Figure 1). The active fraction F4 (722.0 mg) was subjected to preparative HPLC (Phenomenex Luna C18 (2) column; i.d. 250 × 21.2 mm, 10 μm) with MeCN-H_2_O (gradient from 10 to 100% acetonitrile, v/v TFA 0.02%), and then 18 peaks were obtained. The isolated peaks were further purification using semi- preparative HPLC (Phenomenex Luna C18 (2) column; i.d. 250 × 10.0 mm, 5 μm). Peak P5-1 was crystallized from 40% aqueous acetonitrile to give compound **1 **(53.8 mg). Peak P9-2 and P14-2 were purified by semi-preparative HPLC with 30% and 40% acetonitrile isocratic eluents to give compound **2** (0.8 mg) and compound **3** (47.0 mg), respectively. Peak P1518 (58.6 mg) was reseparated by reverse-phase preparative HPLC with acetonitrile-H_2_O (gradient from 40 to 43% acetonitrile for 30 min.) to give compound **6** (6.4 mg). And fraction F6 (200 mg), 2.2 mg of **4** and **5** mixture were obtained by semi-prep HPLC purification (gradient from 10 to 100% acetonitrile for 120 min). Their chemical structures were determined basis on NMR spectral data and MS data and compare with the data of previous reports as: 

**2,3-Dibromo-4,5-dihydroxybenzyl alcohol (lanosol) (1)**. Brown needle crystal; ESIMS *m/z* 296.9 [M − H]^−^ [18].

**2,3-Dibromo-4,5-dihydroxybenzaldehyde (2)**. Colorless solid; ESIMS *m/z* 296.9 [M + H]^+^ [19].

**2,2′,3-Tribromo-3′,4,4′,5-tetrahydroxy-6′-methoxymethyldiphenylmethane (3)**. Colorless solid; ESIMS *m/z* 534.8 [M + Na]^+^ [20].

**2,3-Dibromo-4,5-dihydroxybenzyl methyl ether (4)**. Colorless solid; ESIMS *m/z* 310.9 [M − H]^−^ [18].

**2,5-Dibromo-3,4-dihydroxy-benzyl methyl ether (5)**. Colorless solid; ESIMS *m/z* 310.9 [M − H]^−^ [18].

**2,2′,3,3′-Tetrabromo-4,4′,5,5′-tetrahydroxydiphenylmethane (6)**. White amorphous powder; ESIMS *m/z* 546.7 [M − H]^−^ [20].

### 3.4. Viruses, Cells and Reagents

HRV 2 and 3 were provided by the ATCC (American Type Culture Collection, Manassas, VA, USA) and were propagated in human epitheloid carcinoma cervix (HeLa) cells at 32 °C. HeLa cells were maintained in minimal essential medium (MEM) supplemented with 10% fetal bovine serum (FBS) and 0.01% antibiotic-antimycotic.

Antibiotic-antimycotic, FBS and MEM were supplied by Gibco BRL (Grand Island, NY, USA). The tissue culture plates were purchased from Falcon (BD Biosciences, NJ, USA).

### 3.5. Assays of Antiviral Activity and Cytotoxicity

Assays of antiviral activity and cytotoxicity were evaluated by the SRB method using CPE reduction, recently reported [17]. Briefly, One day before infection, HeLa cells were seeded onto a 96-well culture plate at a concentration of 2 × 10^4^ cells per well. Next day, medium was removed and then washed with 1× phosphate buffered saline (PBS). Infectivity of virus stock was determined by the SRB method using cytopathic effect (CPE) reduction and was determined as infectivity of the virus by SRB ID_50_ (50% infective dose). Following this, 0.09 mL of diluted virus suspension of HRV2 or HRV3 containing CCID_50_ (50% cell culture infective dose) of the virus stock to produce a appropriate cytopathic effects within 2 days after infection and 0.01 mL of medium supplemented with 20 mM MgCl_2_ containing an appropriate concentration of the compounds were added. The antiviral activity of each test material was determined with a 10-fold diluted concentration ranging from 0.1 to 100 μg/mL. Three wells were used as virus controls (virus-infected non-drug-treated cells) while three wells were used as cell controls (non-infected non-drug-treated cells). The culture plates were incubated at 37 °C in 5% CO_2_ for 2 days. After washing 1 times with 1× PBS, 100 μL of cold (−20 °C) 70% acetone were added to each well and left for 30 min at −20 °C. 70% acetone was removed and 96-well plates were left at dry oven for 30 min. 100 μL of 0.4% (w/v) SRB in 1% acetic acid solution were added to each well and left at room temperature for 30 min. Unbound SRB was removed and the plates were washed 5 times with 1% acetic acid before oven drying and were then left in a dry oven for 1 day. Bound SRB was solubilized with 100 μL of 10 mM unbuffered tris-base solution and plates were left on a table for 30 min. The absorbance was read at 540 nm by using a VERSAmax microplate reader (Molecular Devices, Palo Alto, CA, USA) with a reference absorbance at 620 nm. To calculate the IC_50_ values, the results were transformed to percentage of controls and the IC_50_ values were graphically obtained from the dose-response curves. The percent protection achieved by the test compound in HRV2 or HRV3-infected cells was calculated by the following formula: 


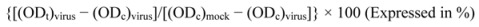
(1)

where (OD_t_)_virus_ is the optical density measured with a given concentration of the test compound in virus-infected cells; (OD_c_)_virus_ is the optical density measured for the control untreated virus-infected cells; and (OD_c_)_mock_ is the optical density measured for control untreated mock-infected cells. The concentration achieving 50% protection according to the formula above was defined as the 50% inhibitory concentration (IC_50_). The therapeutic index was defined as CC_50_/IC_50_. To measure cytotoxicity, HeLa cells were seeded onto a 96-well culture plate at a concentration of 2 × 10^4^ cells per well. Next day, medium was removed and the 96-well plates were replaced with media containing the serially diluted compounds and the cells were further incubated for 48 h. The culture medium was removed and washed with 1× PBS. The next step was conducted by antiviral activity assay above described. To calculate the CC_50_ values, the results were transformed to percentage of controls and the CC_50_ values were graphically obtained from the dose-response curves. Ribavirin was used as positive, and dimethyl sulfoxide (DMSO) was used as negative control.

The effect of compound 1 or compound 3 on HRV-induced CPE was observed. Briefly, HeLa cells were seeded onto a 96-well culture plate at a concentration of 2 × 10^4^ cells per well. The next day, the medium was removed and washed with PBS. Then, 0.09 mL of diluted virus suspension and 0.01 mL of medium supplemented with 20 mM MgCl_2_ containing compound 1 or compound 3 of 20 μg/mL were added. After incubation at 32 °C in 5% CO_2_ for 2 days, the morphology of cells was observed under microscope of 32 × 10 magnifications (St Ernst-Leitz, Wetzlar, Germany), and images were recorded.

## 4. Conclusions

Six polybromocatechols were isolated for the first time from a methanol extract of the red alga *Neorhodomela aculeata*. Two of these compounds, compounds **1** and **3**, exhibited inhibitory activity against human rhinovirus activity in a HeLa cell line. Assuming that these compounds exhibit other drug-like properties, it will be interesting to investigate the preclinical and clinical efficacy of these polybromocatechols.
